# Intravascular ultrasound-guided retrieval of a fish bone embedded in the inferior vena cava: a case report

**DOI:** 10.1186/s42155-026-00778-5

**Published:** 2026-09-29

**Authors:** Leslye F. Conde, Mario Matute-González, Arantxa Gelabert, Adriana Sueiras, Marta Barrufet

**Affiliations:** 1 Vascular and Interventional Radiology Unit, Department of Radiology, Diagnostic Imaging Institute (CDI), Hospital Clínic de Barcelona, Barcelona, Spain; 2 Abdominal Imaging Unit, Department of Radiology, Diagnostic Imaging Institute (CDI), Hospital Clínic de Barcelona, Barcelona, Spain; 3 Clinical Institute of Digestive and Metabolic Diseases (ICMDM), Hospital Clínic de Barcelona, Barcelona, Spain

**Keywords:** Foreign-body migration, Inferior vena cava, Intravascular ultrasound, Endovascular procedures, Venous thrombosis

## Abstract

**Background:**

Intravascular migration of ingested fish bones is an exceptionally rare complication that may result in thrombosis or embolic events. We report a case of inferior vena cava thrombosis caused by fish bone migration successfully managed by IVUS-guided endovascular retrieval.

**Case presentation:**

A 70-year-old woman with stage IV cholangiocarcinoma presented with night sweats, chills, and abdominal pain. Contrast-enhanced CT demonstrated focal inferior vena cava thrombosis containing a linear hyperdense foreign body extending into a renal vein, associated with inflammatory changes adjacent to the duodenum and segmental pulmonary embolism. Fluoroscopic visualization was limited. IVUS enabled precise localization of the foreign body within the thrombus. After unsuccessful snare retrieval attempts, the fish bone was successfully removed using rat-tooth forceps under combined fluoroscopic and real-time IVUS guidance. The patient was discharged asymptomatic on oral anticoagulation planned for 6 months. One-month follow-up CT confirmed complete resolution of inferior vena cava thrombosis and inflammatory changes, with no need for further foreign body-related intervention.

**Conclusions:**

IVUS facilitates retrieval of intravascular foreign bodies embedded within venous thrombus, particularly when fluoroscopic visualization is poor, and can assist in selecting alternative retrieval strategies when conventional snares fail.

## Background

Foreign body ingestion is a common clinical event that is usually resolves with out intervention; however, sharp objects such as fish bones may rarely perforate the gastrointestinal tract and migrate into adjacent structures [1]. Gastrointestinal perforation occurs in less than 1% of cases and may lead to severe vascular complications including thrombosis, pseudoaneurysm formation, hemorrhage, and septic embolization [2].

Venous involvement is particularly uncommon and may present with nonspecific symptoms, making diagnosis challenging [3]. Cross-sectional imaging is essential for diagnosis, whereas endovascular techniques have emerged as minimally invasive alternatives to surgery [3, 4].

We report a case of inferior vena cava (IVC) thrombosis and pulmonary embolism caused by migration of an ingested fish bone, successfully managed with intravascular ultrasound (IVUS)-guided endovascular retrieval.

## Case presentation

A 70-year-old woman with stage IV cholangiocarcinoma undergoing gemcitabine chemotherapy presented to the outpatient clinic with a 1-month history of night sweats, chills, and right iliac fossa pain. Previous treatment with cefixime for urinary tract infection and amoxicillin–clavulanate for gingival inflammation had failed to improve symptoms.

**Fig. 1:**
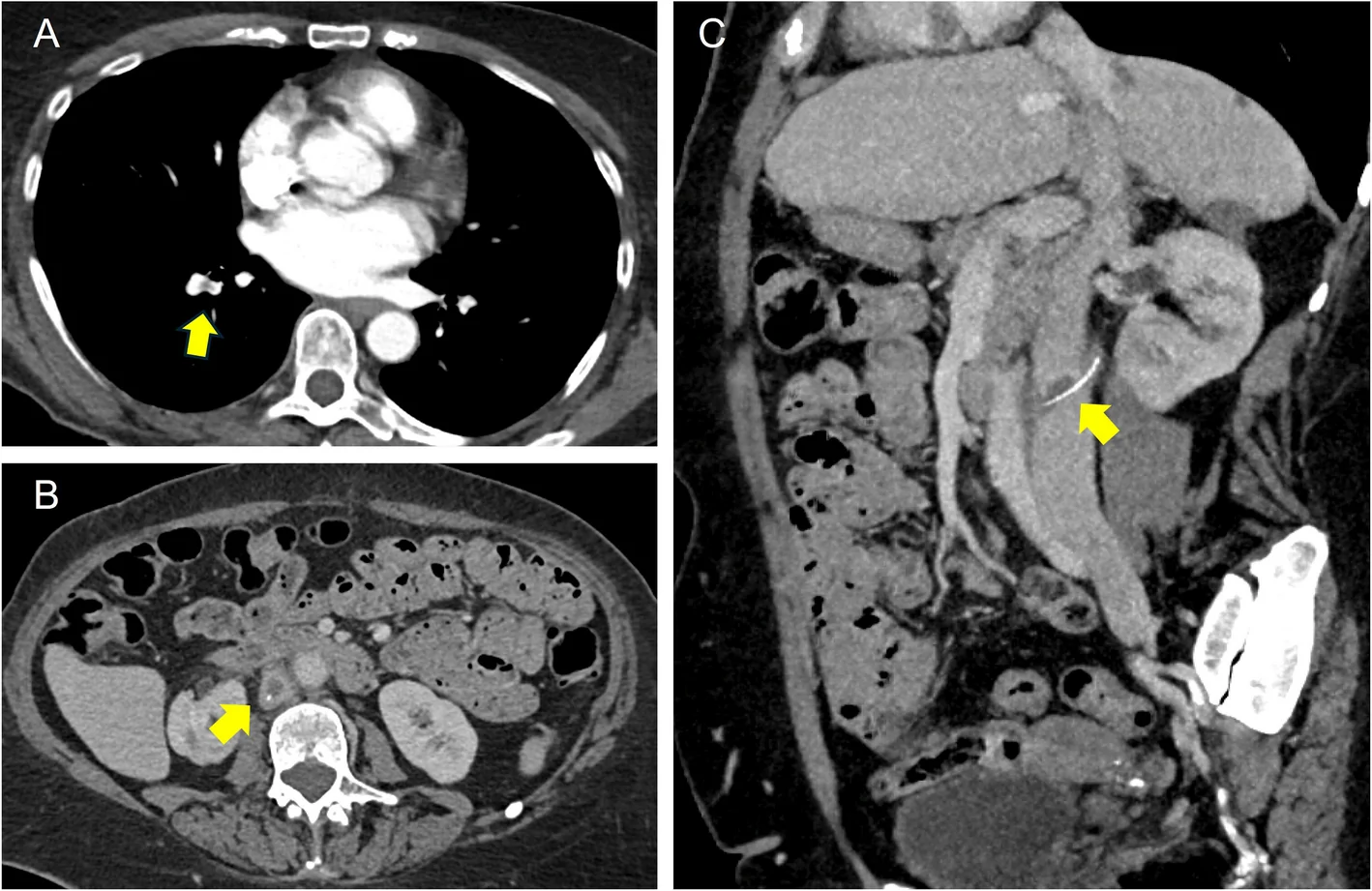
CT findings of the intracaval fish bone and associated complications. **A** CT pulmonary angiography showing a segmental pulmonary embolism in the right lower lobe (arrow). **B** Axial contrast-enhanced CT demonstrating a linear hyperdense foreign body within the inferior vena cava associated with focal non-occlusive thrombosis (arrow) (**C**) Sagittal MPR-MIP reconstruction showing the fish bone extending toward the right renal vein (arrow)

Contrast-enhanced CT performed for oncologic follow-up demonstrated focal nonocclusive thrombosis of the infrarenal IVC containing a linear hyperdense foreign body compatible with a fish bone, with one extremity extending into a small right renal vein. Marked interaortocaval inflammatory fat stranding adjacent to the third portion of the duodenum was also identified, consistent with the presumed route of transmural migration of the fish bone into the IVC. Additionally, a small segmental pulmonary embolism was present (Fig. 1).

Following the CT findings, the patient was specifically questioned about possible fish bone ingestion. She then recalled having recently increased her fish consumption but did not remember swallowing a fish bone or experiencing any choking episode.

The patient was admitted and started on intravenous piperacillin–tazobactam, daptomycin, and therapeutic anticoagulation. Despite previous outpatient antibiotic therapy and subsequent broad-spectrum intravenous antibiotics, she continued to experience malaise, night sweats, and chills, with persistent leukocytosis and elevated C-reactive protein levels. Given the persistent systemic inflammatory syndrome, the presence of pulmonary embolism, and the retained intravascular foreign body with its ongoing risk of infectious and thromboembolic complications, conservative management alone was considered insufficient. After multidisciplinary discussion, endovascular retrieval was therefore undertaken.

**Fig. 2:**
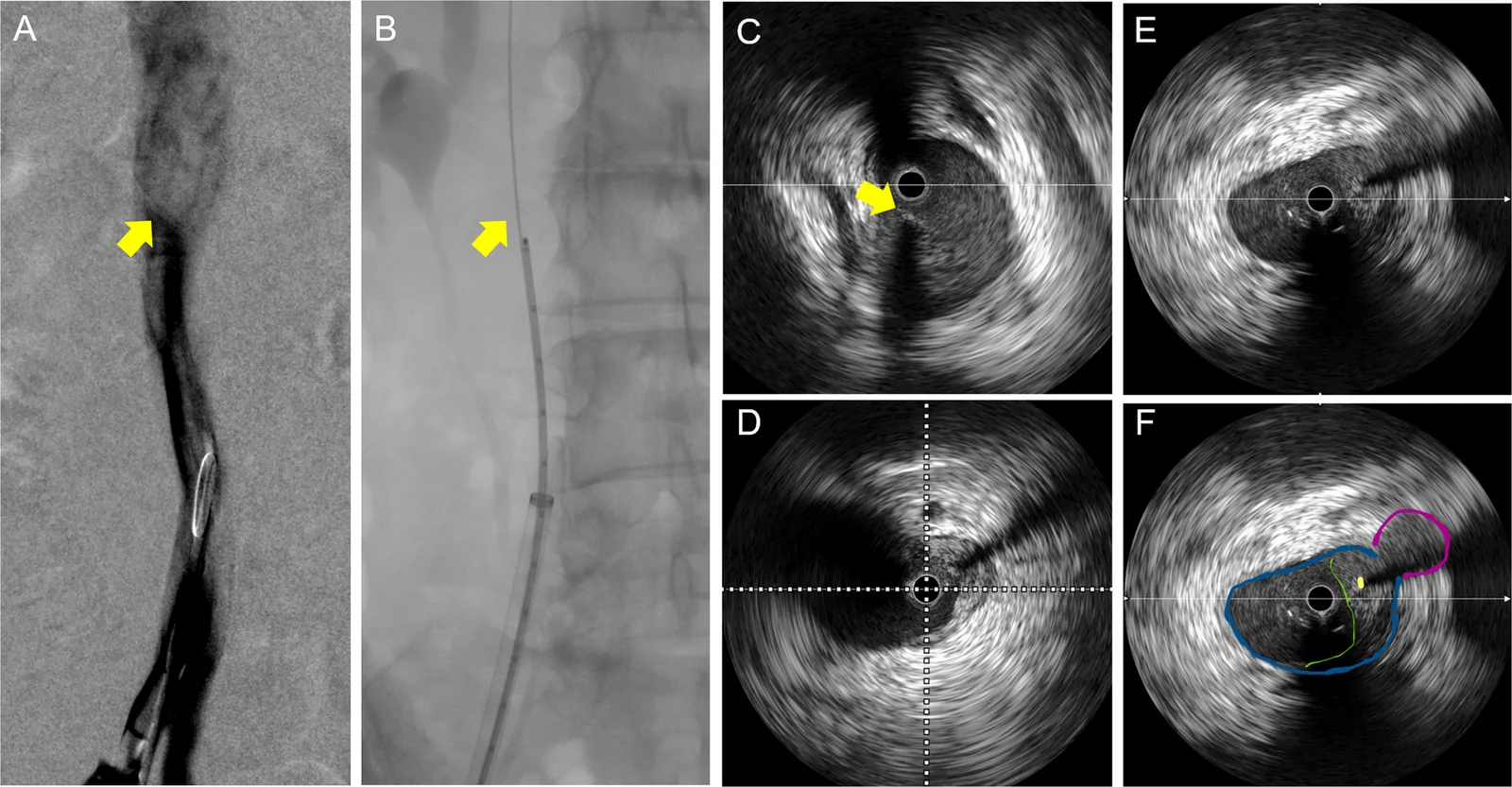
Venography and IVUS findings. **A** Initial IVC venography showing only a subtle filling defect (arrow) at the level of L3. **B** Under fluoroscopy guidance, the IVUS catheter was positioned at the level of previously identified foreign body. **C**, **D** Cranial-to-caudal IVUS examination revealing a hyperechogenic linear foreign body with posterior acoustic shadowing embedded within focal thrombosis. **E**, **F** IVUS image and corresponding schematic overlay depicting the fish bone (yellow) embedded within mural thrombus (green) in the IVC (blue), adjacent to the renal vein (purple)

Initial IVC venography with digital subtraction angiography (DSA) via right femoral venous access was performed using an Artis Q angiography system (Siemens Healthineers, Forchheim, Germany) and demonstrated only a subtle filling defect. Despite the use of DSA, the foreign body remained poorly visualized under fluoroscopy, becoming barely perceptible only after contrast injection. In contrast, IVUS clearly demonstrated a hyperechogenic linear structure with posterior acoustic shadowing embedded within focal caval thrombosis and extending toward a renal vein (Fig. 2). IVUS enabled identification of the free intraluminal tip of the foreign body, facilitating targeted retrieval.

To reduce the risk of embolization during retrieval, a suprarenal IVC filter (Cook Celect Platinum, Cook Medical, Bloomington, IN, USA) was deployed through an 8-Fr right internal jugular vein access. Under systemic anticoagulation with intravenous heparin (5000 IU; activated clotting time = 200 s), an OptiCross 35 IVUS catheter (Boston Scientific, Marlborough, MA, USA) was advanced through the jugular access to localize the foreign body and assess its orientation relative to the surrounding thrombus.

**Fig. 3:**
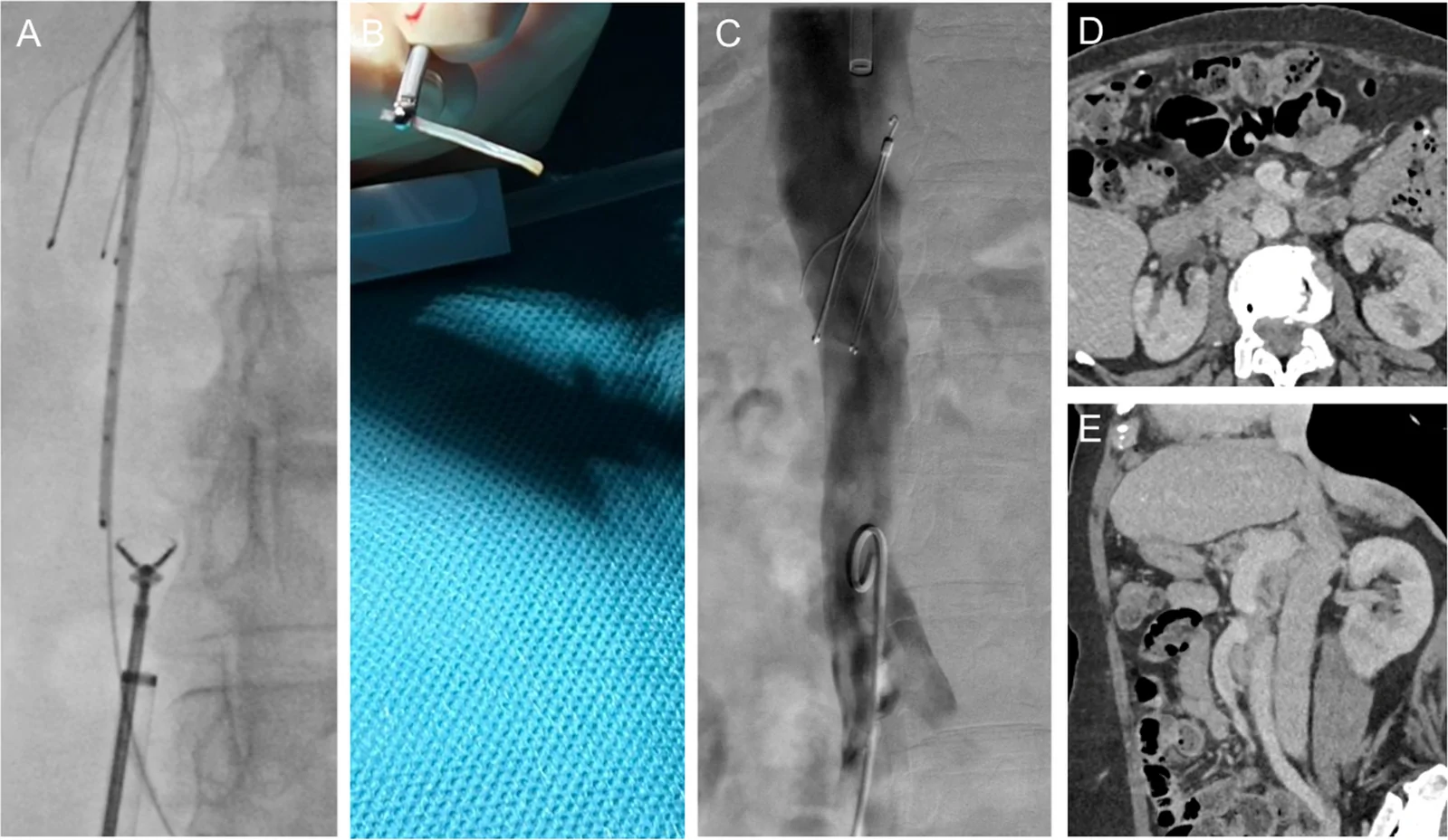
Endovascular retrieval and follow-up. **A** Following deployment of a temporary suprarenal IVC filter, rat-tooth forceps were used to successfully retrieve the fish bone. **B** Extracted fish bone. **C** Completion venography showing no residual filling defects. **D**, **E** Follow-up contrast-enhanced CT at 1 month demonstrating complete removal of the foreign body and resolution of the associated thrombosis

After upsizing the femoral venous access to a 12-Fr sheath, retrieval was initially attempted using a 20-mm single-loop snare (ONE Snare, Merit Medical, South Jordan, UT, USA), followed by a triple-loop snare (En Snare, Merit Medical, South Jordan, UT, USA). A handmade snare, fashioned from a 0.035-inch guidewire advanced through a Simmons diagnostic catheter, was subsequently used. None of these techniques successfully engaged the foreign body, and IVUS demonstrated no displacement following repeated retrieval attempts. Subsequently, a rat-tooth biopsy forceps (Radial Jaw 4 Biopsy Forceps, Boston Scientific, Marlborough, MA, USA) was introduced through the sheath. Under combined fluoroscopic and real-time IVUS guidance, the free intraluminal tip of the foreign body was successfully grasped and removed (Fig. 3). The retrieved object was confirmed to be a fish bone.

Final IVUS evaluation demonstrated residual mural caval thrombosis without procedural complications, and completion venography showed no residual filling defects. The IVC filter was then retrieved.

The day after the intervention, the patient reported complete resolution of symptoms. She was discharged asymptomatic after completing the prescribed antibiotic course, with no procedure-related complications, and was maintained on therapeutic oral anticoagulation for a planned total duration of 6 months. At 1-month follow-up, CT demonstrated complete resolution of inferior vena cava thrombosis and the surrounding inflammatory changes. No further intervention or imaging related to the foreign body was deemed necessary. The patient continued routine oncologic follow-up for her underlying malignancy while remaining asymptomatic from the vascular complication.

## Discussion

Most ingested foreign bodies pass spontaneously through the gastrointestinal tract without clinical consequences, whereas only a small proportion results in perforation. The estimated incidence of fish bone–related bowel perforation is approximately 2.9 per 100,000 person-years, underscoring the rarity of clinically significant complications [1, 4, 5]. Patients may remain asymptomatic for prolonged periods or present with chronic, nonspecific symptoms such as night sweats, chills, and localized abdominal pain, as observed in this case [3, 6].

Nevertheless, once perforation occurs, migration beyond the gastrointestinal tract may lead to a wide spectrum of extra-digestive complications. A review of 107 cases of migrating fish bones and toothpicks demonstrated that the peritoneal cavity represents the most common site of migration, accounting for 64.5% of cases, further emphasizing the exceptional nature of vascular involvement [7].

The pathophysiology of vascular complications is not fully understood but is generally attributed to progressive transmural erosion following perforation. A combination of local inflammatory response, peristalsis, and body movements may facilitate migration and eventual penetration into adjacent vascular structures [5, 6, 8].

Arterial complications usually manifest as pseudoaneurysm formation or life-threatening hemorrhage and frequently require urgent endovascular treatment [9–11]. In contrast, venous involvement is distinctly uncommon and follows a different clinical and pathophysiological pattern. Rather than acute hemorrhage, venous injury is more frequently associated with thrombosis, septic embolization, or silent intravascular migration [12]. As reported in the literature, foreign body–induced perforation may trigger localized inflammation and a prothrombotic state, predisposing to venous thrombosis in adjacent vascular territories [3, 12, 13]. Clinical presentation is often insidious, and diagnosis is typically established through cross-sectional imaging, where the fish bone can be seen as a calcified linear structure [4].

In such cases, endovascular retrieval represents a minimally invasive and effective therapeutic strategy. Technical challenges arise particularly when the foreign body is poorly visible under fluoroscopy or partially embedded within thrombus [1, 11].

In this context, IVUS provided a major procedural advantage by allowing real-time visualization of the foreign body despite its limited fluoroscopic conspicuity. IVUS enabled precise localization and orientation of the fish bone, as well as assessment of its relationship to the vessel wall and surrounding thrombus. This facilitated targeted retrieval and potentially reduced the risk of embolization or vascular injury [3].

Although loop snares remain the most commonly used retrieval devices, their effectiveness may be limited by morphology, orientation, and degree of embedding of the foreign body. In such scenarios, alternative tools, such as rat-tooth or alligator biopsy forceps, may be useful. Although their indovascular use is off-label, biopsy forceps have demonstrated high technical success and low complication rates in large series of IVC filter retrieval [14, 15]. The main concern is inadvertent grasping of the venous wall, with potential perforation or pseudoaneurysm formation. In the present case, this risk was mitigated by real-time IVUS confirmation of the free intraluminal tip before grasping, and careful manipulation through a 12-Fr sheath under continuous combined IVUS and fluoroscopic guidance, enabling direct extraction of the fish bone.

Compared with previously published reports, which predominantly describe arterial complications or fluoroscopy-guided retrieval, the present case illustrates several distinctive features: venous involvement of the IVC with extension into a renal vein, associated thrombosis and pulmonary embolism, use of combined fluoroscopic and IVUS guidance, and successful retrieval using endoscopic forceps. Given the potential risk of embolization during manipulation, a temporary suprarenal IVC filter was used as a protective measure [3]. These findings highlight the importance of adapting the endovascular strategy to the anatomical and mechanical characteristics of the foreign body.

Although endovascular retrieval was successful, surgical removal would likely have been the next therapeutic option had percutaneous techniques failed, as conservative management would have left the retained foreign body in situ, with persistent risks of infection, thromboembolic complications, and ongoing inflammation. In this setting, off-label use of endoscopic biopsy forceps provided an effective bailout strategy after failed snare retrieval under continuous IVUS and fluoroscopic guidance.

This report has several limitations. As a single case, it does not allow comparison with alternative management strategies or establish the superiority of IVUS-guided retrieval over conventional fluoroscopic techniques. Given the extreme rarity of intravascular fish bone migration, multicenter registries and additional case reports will be required to better define patient selection, procedural strategies, and long-term outcomes.

## Conclusion

Intravascular migration of fish bones is exceptionally rare but may result in serious thromboembolic complications. In selected patients, IVUS-guided endovascular retrieval using grasping forceps represents a feasible and minimally invasive alternative when fluoroscopic visualization is limited. Further experience is required to define the role of IVUS in complex intravascular foreign body retrieval.

## Data Availability

All data generated or analyzed during this study are included in this published article.
